# SARS-CoV-2 replicates in the human testis with slow kinetics and has no major deleterious effects *ex vivo*

**DOI:** 10.1128/jvi.01104-23

**Published:** 2023-10-13

**Authors:** Dominique Mahé, Salomé Bourgeau, Janaina da Silva, Julie Schlederer, Anne-Pascale Satie, Nadège Kuassivi, Romain Mathieu, Yves-Marie Guillou, Anna Le Tortorec, Florence Guivel-Benhassine, Olivier Schwartz, Ingrid Plotton, Nathalie Dejucq-Rainsford

**Affiliations:** 1 Institut National de la Santé et de la Recherche Médicale, Ecole des Hautes Etudes en Santé Publique, Institut de recherche en santé, environnement et travail, Université de Rennes, UMR_S1085, Rennes, France; 2 University of CAS, Beijing, China; 3 CAS Key Laboratory of Molecular Virology & Immunology, Institut Pasteur of Shanghai CAS, Shanghai, China; 4 Service d‘Urologie, Centre Hospitalier Universitaire de Rennes, Rennes, France; 5 Service de Coordination des prélèvements, Centre Hospitalier Universitaire de Rennes, Rennes, France; 6 Virus and Immunity Unit, Institut Pasteur, Université de Paris Cité, CNRS UMR3569, Paris, France; 7 Institut National de la Santé et de la Recherche Médicale, Institut Cellules Souche et Cerveau (SBRI), UMR_S1208, Bron, France; Loyola University Chicago, Maywood, Illinois, USA

**Keywords:** SARS-CoV-2, pathogenesis, innate response, target cells, testis, *ex vivo* model

## Abstract

SARS-CoV-2, the etiological agent of the ongoing Covid-19 pandemic, is a respiratory virus that infects several organs beyond the lungs. The alterations of semen parameters, testicular morphology, and testosteronemia reported in Covid-19 patients, along with the high expression of SARS-CoV-2 main receptor ACE2 in the testis, raise questions about the underlying mechanisms. Using a previously validated *ex vivo* model of human testis, we show that SARS-CoV-2 infects ACE2-positive Leydig and Sertoli cells. Slow virus replication kinetics were observed, with a peak of infection at day 6 post-infection and a decline of infectious virions at day 9. The infection had no major impact on testicular morphology or main hormonal functions, but some steroidogenic enzymes were decreased at day 9. While antiviral effectors were upregulated, there was no transcriptional induction of key pro-inflammatory cytokines. Altogether, these data indicate that SARS-CoV-2 replication in the human testis *ex vivo* is limited and suggest that testicular damages in infected individuals are unlikely to result from direct deleterious effects of SARS-CoV-2 on this organ.

**IMPORTANCE:**

SARS-CoV-2 is a new virus responsible for the Covid-19 pandemic. Although SARS-CoV-2 primarily affects the lungs, other organs are infected. Alterations of testosteronemia and spermatozoa motility in infected men have raised questions about testicular infection, along with high level in the testis of ACE2, the main receptor used by SARS-CoV-2 to enter host cells. Using an organotypic culture of human testis, we found that SARS-CoV-2 replicated with slow kinetics in the testis. The virus first targeted testosterone-producing Leydig cells and then germ-cell nursing Sertoli cells. After a peak followed by the upregulation of antiviral effectors, viral replication in the testis decreased and did not induce any major damage to the tissue. Altogether, our data show that SARS-CoV-2 replicates in the human testis to a limited extent and suggest that testicular damages in infected patients are more likely to result from systemic infection and inflammation than from viral replication in the testis.

## INTRODUCTION

SARS-CoV-2, the causative agent of the ongoing Covid-19 pandemic, is a new beta-coronavirus first identified in China in December 2019. SARS-CoV-2 has rapidly disseminated worldwide and infected millions of people, generating a high death toll and long-term health damages. Although this emerging virus is primarily a respiratory virus, its pathogenicity extends beyond the lungs, with notable alterations of the gastro-intestinal and uro-genital tracts (1 – 3). It is now clear that SARS-CoV-2 can spread to different organs, either through early viremia or infection of the vasculature (2, 4, 5). Studies in organoids and/or in deceased patients demonstrated that SARS-CoV-2 infects tissues such as kidney (6, 7), capillary (6), gastro-intestinal organs (8, 9), and brain (4). ACE2, the main receptor used by SARS-CoV-2 to enter host cells, is highly expressed in the testis, suggesting that the testes may be at risk of infection (10 – 12).

In SARS-CoV-2-infected patients, alterations of semen parameters (e.g., decreased sperm count and mobility) (13 – 19) and male hormones (13, 20 – 22) have been reported in many studies, along with damaged testicular morphology in deceased individuals (degenerated germ cells, reduced Leydig cell numbers, damaged Sertoli cells, and inflammatory infiltrates) (3, 13, 19, 23). Whether these alterations result from a direct infection of the testis or from the systemic infection and high level of inflammation is unknown. Most studies except a few (15, 16, 24) have failed to detect SARS-CoV-2 in semen, indicating that seminal excretion is rare (19). Nonetheless, SARS-CoV-2 nucleic acids and proteins have been evidenced in the testes of deceased patients (70/136 in 9 studies) (2, 4, 13, 23, 25 – 27), suggesting that this organ is a target for SARS-CoV-2. However, autopsy data often suffer from issues such as blood contaminations, technique specificity, altered tissue morphology, poor tissue and viral nucleic acid preservation, and/or windows of tissue collection distant from acute infection (28). A recent *in vitro* study failed to detect productive infection of different isolated testicular cell types in 2D or 3D cultures (29). Whether SARS-CoV-2 can replicate in the human testis tissue and directly alter the organ’s functions and morphology remains unclear. Testicular infection has been reported *in vivo* in animal models to various extents depending on the studies and species (transgenic mouse, Syrian hamsters, and macaques) (30 – 35). However, the tropism of SARS-CoV-2 for the human testis cannot be extrapolated from these findings, notably because of distinct characteristics between animal and human testes (e.g., differences in innate immune responses and cell and morphological specificities) (36 – 38).

*Ex vivo* studies have been critical for uncovering the organ and cell tropism of SARS-CoV-2 in humans (1, 39). Here, we used our original *ex vivo* model of human testis—which relevance to infer testis tropism *in vivo* has been validated for a range of viruses (36, 40, 41)—to determine whether SARS-CoV-2 replicates in this organ, decipher its target cells, and assess the impact of the infection on the testis’ innate immune responses and functions.

## RESULTS

### SARS-CoV-2 replicates with slow kinetics in the human testis *ex vivo*, spreading from the interstitial tissue to the seminiferous tubules

**Fig 1:**
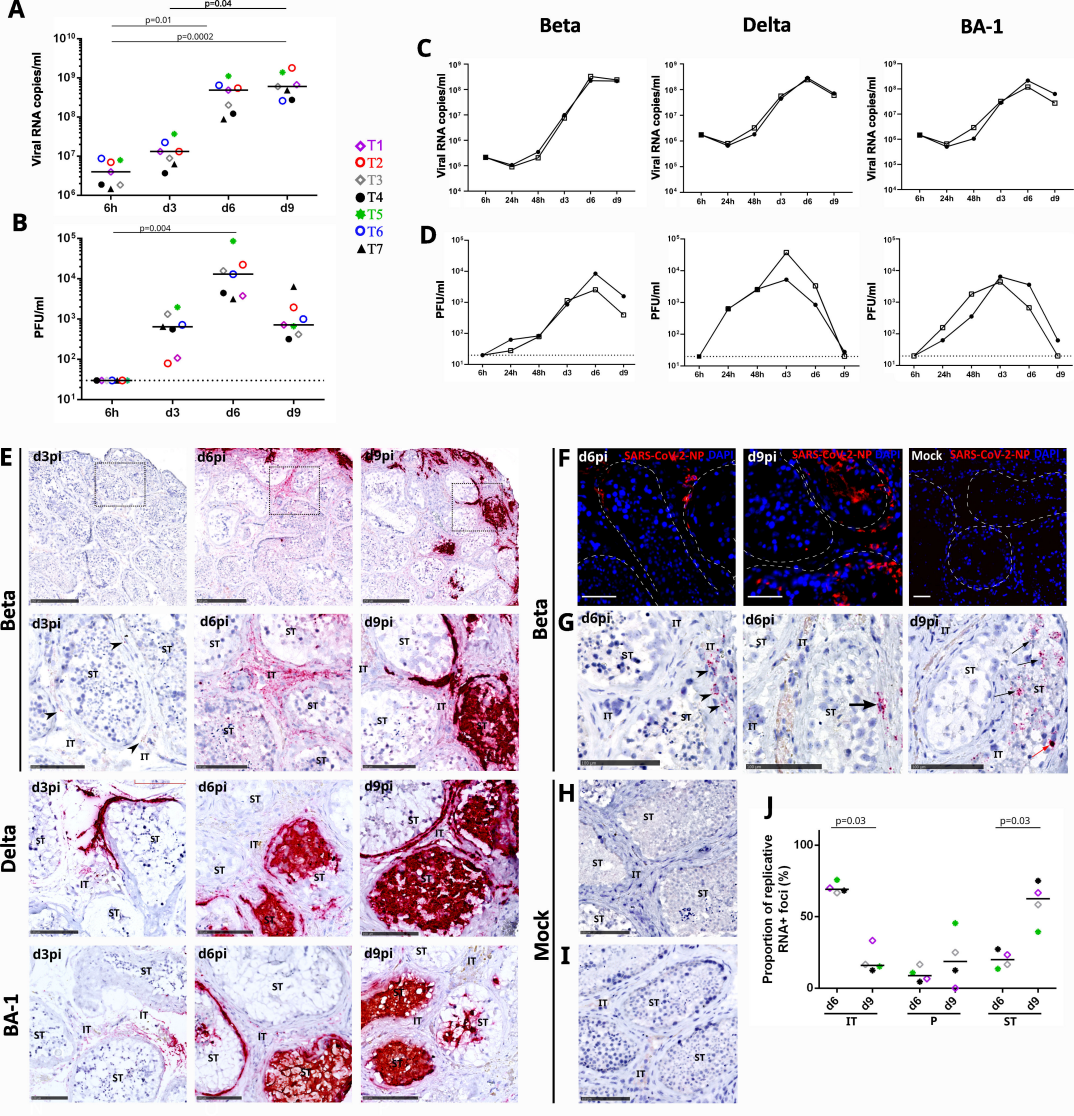
SARS-CoV-2 replicates in human testis explants and spreads from interstitial tissue to seminiferous tubules. (**A and B**) Human testis explants from seven testis donors were infected overnight at a multiplicity of infection (MOI) of 1 (corresponding to 7·10^4^ PFU and 3·10^8^ vRNA copies) with a low-passage SARS-CoV-2 beta strain isolated in 2020 in France (IDF0372_2020_C2). Explants were thoroughly washed and cultured on inserts with 1-mL medium per well for 9 days. Media were fully removed and changed every 3 days. Supernatants were harvested at 6 h (set as baseline value for assessing residual inoculated virus after incubation and washes), d3, d6, and d9 pi. Each of the time points (days 3, 6, and 9), therefore, represents *de novo* viral release over a 3-day-culture period. Each symbol represents the median value of all infected wells for a defined donor (same symbol throughout the figures). Horizontal bars represent the median values for all donors. Statistical analyses were performed using Friedman-Dunn non-parametric comparison. (**C and D**) The replication of SARS-CoV-2 delta, omicron BA-1, and beta variants was compared in an additional testis donor using the same protocol as above. The results for the two independent wells tested are shown. (**A and C**) SARS-CoV-2 RNA levels were quantified by reverse transcripase-quantitative polymerase chain reaction (RT-qPCR) against ORF1b-nsp14 mRNA in culture supernatants. vRNA was consistently below detection threshold in mock-infected explant supernatants. (**B and D**) Viral titers were measured by infectivity assay on VeroE6 cells. Mock-infected explants were all negative. (**E and H**) RNAScope *in situ* hybridization (ISH) was undertaken using a probe targeting SARS-CoV-2 genomic RNA in testis explants infected with SARS-CoV-2 beta (broad and zoomed views of the framed area are shown), delta, or omicron BA-1 strains at day 3, 6, and 9 post-infection (**E**) versus mock infected (**H**). (**F**) Immunohistochemistry using a specific antibody against the SARS-CoV-2 nucleoprotein (NP) was performed on testis explants exposed to SARS-CoV-2 beta strain at day 6 and day 9 post-infection. No staining was ever observed in mock-infected testis explants. Nuclei are stained in blue. Scale bars, 50 µm. (**G and I**) Representative images of RNAScope ISH using a probe targeting SARS-CoV-2 replicative RNA at day 6 and 9 post-infection with beta strain (**G**) and in control mock-infected testis explants (**I**). Black arrow heads point at infected cells in the interstitial tissue. Thick black arrow points at infected cells in the extracellular matrix surrounding the seminiferous tubules. Thin black arrows point at infected cells in the seminiferous tubules. The thin red arrow points at an infected germ cell. Scale bars, 100 µm. (**J**) The number of infected cell foci in the interstitial tissue (IT), peritubular connective tissue surrounding seminiferous tubules (**P**), and number of infected seminiferous tubules (ST) were quantified in at least three whole testis sections (four donors, beta strain infection) labeled with SARS-CoV-2 replicative RNA on day 6 and 9 pi. *P < 0.05 (Mann-Whitney non-parametric comparison).

Testis explants from seven uninfected donors (age range, 29–69) were exposed to SARS-CoV-2 BetaCoV strain, as we described for Zika virus (36). vRNA levels increased from a median of 4,01·10^6^ copies/mL at baseline (corresponding to 6 h after inoculum removal and washes) to a median of 1,32·10^7^ copies/mL (range, 6,27·10^6^–3,74·10^7^) at 3 days post-infection (dpi) and 5,21·10^8^ copies/mL (range, 8,89·10^7^–1,12·10^9^) at 6 dpi (Fig. 1A). At 9 dpi, vRNA stabilized at a median of 6,4·10^8^ copies/mL (range, 2,63·10^8^–1,82·10^9^). The cumulated vRNA released over the 9-day culture period reached a median of 1,49·10^9^ copies/well (range, 4,51·10^8^–2,38·10^9^), above viral inoculum (9,97·10^7^ copies/well) (Fig. 1A). SARS-CoV-2 viral titer in testis explant supernatants significantly increased from undetectable at baseline [6 hours post-infection (hpi)] to a median of 6,84·10^2^ PFU/mL (range, 8·10^1^–1,97·10^3^) at 3 dpi and 1,44·10^4^ PFU/mL (range, 3,16·10^3^–8,63·10^4^) at 6 dpi, whereas it decreased to 8,6·10^2^ PFU/mL (range, 4,2·10^2^–6,4·10^3^) at 9 dpi (Fig. 1B). The cumulated PFU released during the 9-day culture period ranged from 4,63·10^3^ to 8,89·10^4^/well (viral inoculum, 3,5·10^4^ PFU/well). These results demonstrate that SARS-CoV-2 beta strain slowly replicated in human testis explants to reach a peak of infectious virions production in between day 3 and 6 post-infection, while viral titer declines thereafter. Since other SARS-CoV-2 strains appeared and disappeared since the start of our study, we compared the replication of delta, omicron BA-1, and beta variants in order to assess the generalization of the beta strain data. Consistent with our above data, the three strains showed a peak of vRNA at day 6 in testis explants (Fig. 1C). A peak of infectious virion production followed by a decline in viral titer occurred at day 3 for delta and omicron BA-1 variants (mean of 2,15·10^4^ and 5,42·10^3^ PFU/mL, respectively) versus day 6 for SARS-CoV-2 beta strain (5,52·10^3^ PFU/mL) (Fig. 1D).

*In situ* detection of SARS-CoV-2 genomic RNA (Fig. 1E), structural nucleoprotein (Fig. 1F), and replicative RNA (Fig. 1G) was undertaken on SARS-CoV-2-infected and mock-infected testis explants, using *in situ* hybridization (ISH) (RNAscope) and immunofluorescence. Genomic vRNA labeling detected by RNAscope increased over time in testis explants infected with either SARS-CoV-2 beta, delta, or omicron BA-1 strains, with staining progressing from the interstitial tissue to the seminiferous tubules at days 6 to 9 (Fig. 1E). The progression of infection within the testis tissue was also evidenced using antibodies against SARS-CoV-2 nucleoprotein (Fig. 1F). Viral replicative RNA detection and quantification were performed in testis explants exposed to SARS-CoV-2 beta strain (Fig. 1G). Foci composed of one or several productively infected cells were detected in the interstitial tissue and close to the seminiferous tubules lined by peritubular cells at 6 dpi (Fig. 1G). The number of interstitial foci decreased at 9 dpi, whereas the number of seminiferous tubules harboring replicative vRNA increased (Fig. 1G and J). The staining for viral replicative RNA at days 6 and 9 was weaker and less widespread than for genomic RNA. Together with the progressive infection inside the tubules and parallel viral titer decline from d6, this suggests abortive infection in a proportion of testicular cells.

### SARS-CoV-2 primarily targets ACE2-positive Leydig cells and Sertoli cells in testis explants

**Fig 2:**
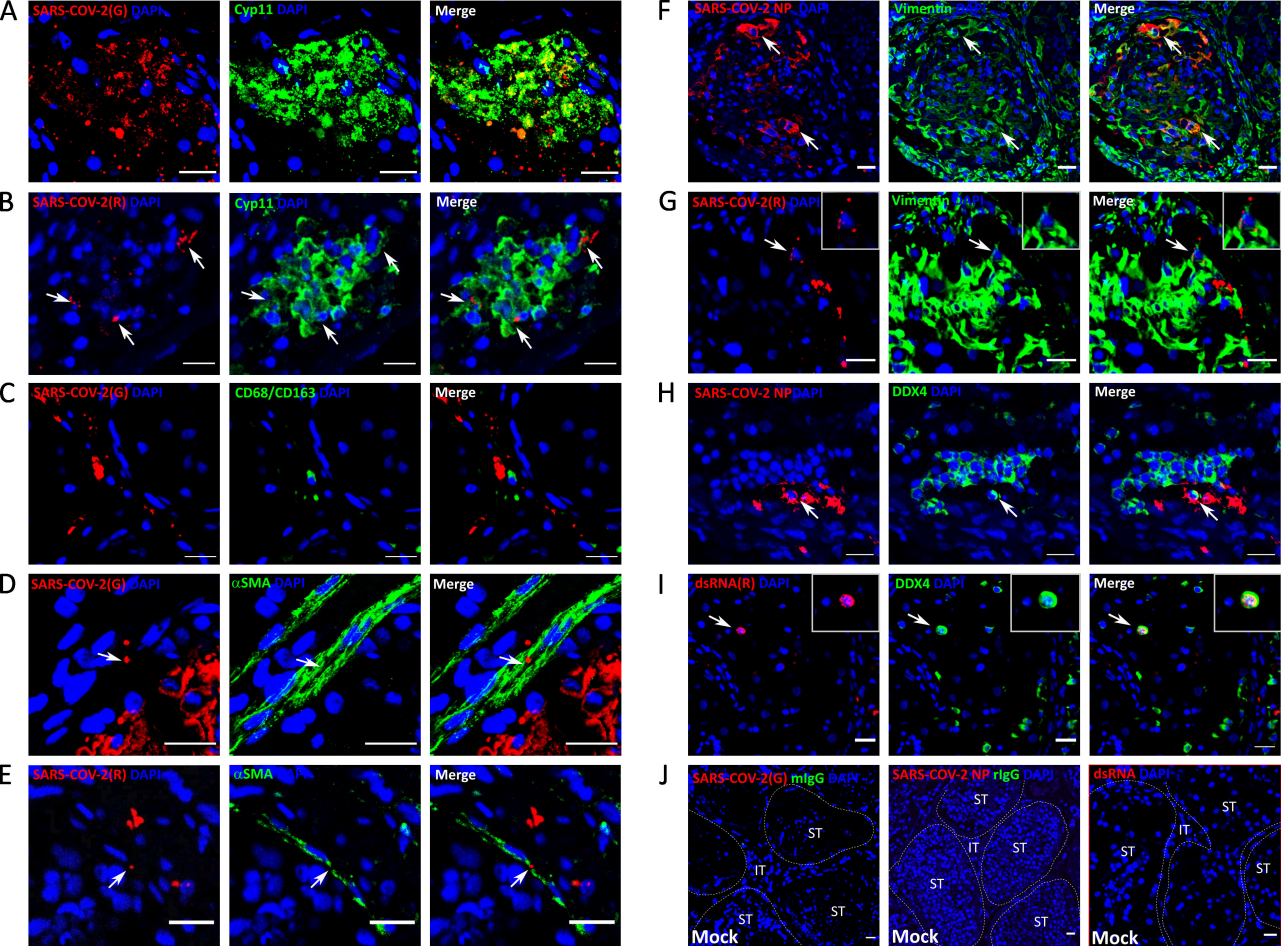
Identification of SARS-CoV-2-infected cells in human testis explants. RNAscope ISH using probes targeting (**A, C, and D**) SARS-CoV-2 genomic (**G**) RNA or (**B, E, and G**) SARS-CoV-2 replicative (**R**) RNA was coupled with immunofluorescence for cell markers to label (**A and B**) Cyp11A1+ Leydig cells, (**C**) CD68/CD163+ macrophages, (**D and E**) αSMA+ peritubular cells, (**F and G**) vimentin+ Sertoli cells, or (**H and I**) DDX4+ germ cells in SARS-CoV-2 beta strain-infected explants. (**F and H**) IHC against SARS-CoV-2 nucleoprotein (NP) or (**I**) double-stranded RNA (dsRNA) was undertaken to further characterize infected cells in the seminiferous tubules. (**J**) No staining for SARS-CoV-2 by ISH or IHC and for cell marker isotypes was ever observed in mock-infected testis. Nuclei are stained in blue. Scale bars, 20 µm.

To characterize the infected cell types, RNAscope for both genomic and replicative SARS-CoV-2 RNA was combined with fluorescent immunohistochemistry (IHC) against specific cell markers in testis explants. Immunofluorescence using antibodies against SARS-CoV-2 structural nucleoprotein or replicative double-stranded RNA was undertaken as a complementary approach. In the interstitial compartment of testis explants exposed to SARS-CoV-2 beta strain, Cyp11A1+ Leydig cells stained positive for both SARS-CoV-2 genomic (G) and replicative (R) RNA at day 6 and showed internalized replicative RNA (Fig. 2A and B; Fig. S1A). In contrast, SARS-COV-2 RNA never colocalized with CD68+/CD163+ resident macrophages (Fig. 2C). α-SMA+ peritubular myoid cells surrounding the seminiferous tubules only occasionally stained positive for SARS-CoV-2 genomic and replicative RNA (Fig. 2D and E; Fig. S1B). In the seminiferous tubules, the large labeling for SARS-CoV-2 genomic RNA—observed essentially at day 9 for the beta strain and from day 6 onwards for delta and omicron—tended to obstruct cell marker detection by IHC. We, therefore, favored immunofluorescence against NP (Fig. 2F and H) or dsRNA (Fig. 2I) as well as RNAscope for replicative RNA (Fig. 2G; Fig. S1C) for co-localization. Vimentin+ Sertoli cells harboring SARS-CoV-2 proteins and internalized replicative vRNA were detected at 9 dpi in SARS-CoV-2 beta strain-infected explants (Fig. 2F and G; Fig. S1C). Only rare isolated DDX4+ germ cells were infected at 9 dpi, as observed with SARS-CoV-2 proteins and double-stranded RNA labeling (Fig. 2H and I). SARS-CoV-2 delta and omicron BA-1 strains also primarily infected Leydig cells and Sertoli cells in testis explants, along with peritubular cells (Fig. S2).

In line with the scarce detection of infected testicular germ cells, the exposure of germ cells freshly isolated from human testis to SARS-CoV-2 beta strain did not lead to infectious titer increase during the 4 days of *in vitro* culture. Only rare primary DDX4+ germ cells stained positive for double-stranded RNA, indicating that germ cells are not a key target for SARS-CoV-2 (Fig. S3).

The expression in the testis of the host receptors and proteases described or suspected to mediate SARS-CoV-2 entry was investigated using RNA sequencing (RNA-seq) databases and RT-qPCR. RNA-seq data from four distinct studies (42 – 45) (Fig. S4A) and RT-qPCR on testis explants (Fig. S4B) concordantly showed the expression of ACE2 and alternative receptors BSG, AXL, and NRP1 mRNAs in the testis, whereas TIM-1 was little expressed. TMPRSS2 was below RT-qPCR detection threshold in three out of five donors and showed low expression in all RNA-seq studies. Furin was detected in RNA-seq and was above RT-qPCR detection threshold in three out of five testis donors. The proteases CTSL and CTSB transcripts were readily measured (Fig. S4B). ACE2 protein localization was examined using IHC in the testis explants of four donors (age range, 18–51). ACE2 staining colocalized with Cyp11A1+ Leydig cells in the interstitial tissue and bordering the seminiferous tubules (Fig. S5A), as well as with vimentin+ Sertoli cells inside the seminiferous tubules (Fig. S5D). In contrast, ACE2 was not detected in CD68/CD163+ macrophages (Fig. S5B) nor in SMA+ peritubular myoid cells (Fig. S5C). The vast majority of germ cells did not express ACE2 (Fig. S5E), but a few DDX4+ germ cells stained positive (Fig. S5F). The expression of ACE2 mRNA and ACE2 protein localization was unchanged in infected versus uninfected testis explants during the culture period and was similar across patients, irrespective of their age (Fig. S5H and S6A and B). Consistent with RNA-seq and RT-qPCR data, TMPRSS2 protein expression in the testis was undetectable using IHC on testis tissue from all donors tested (Fig. S6C).

In summary, our results indicate that SARS-CoV-2 primarily infects ACE2+ Leydig cells within the testis interstitial tissue *ex vivo*, before spreading into the seminiferous tubules, in which ACE2+ Sertoli cells represent the main targets. SARS-CoV-2 was also detected in some myoid peritubular cells and rare isolated testicular germ cells but was never associated with testicular macrophages, which were negative for ACE2 protein.

### Low-level induction of antiviral response in SARS-CoV-2-infected testis explants precedes the decline in infectious virions release

**Fig 3:**
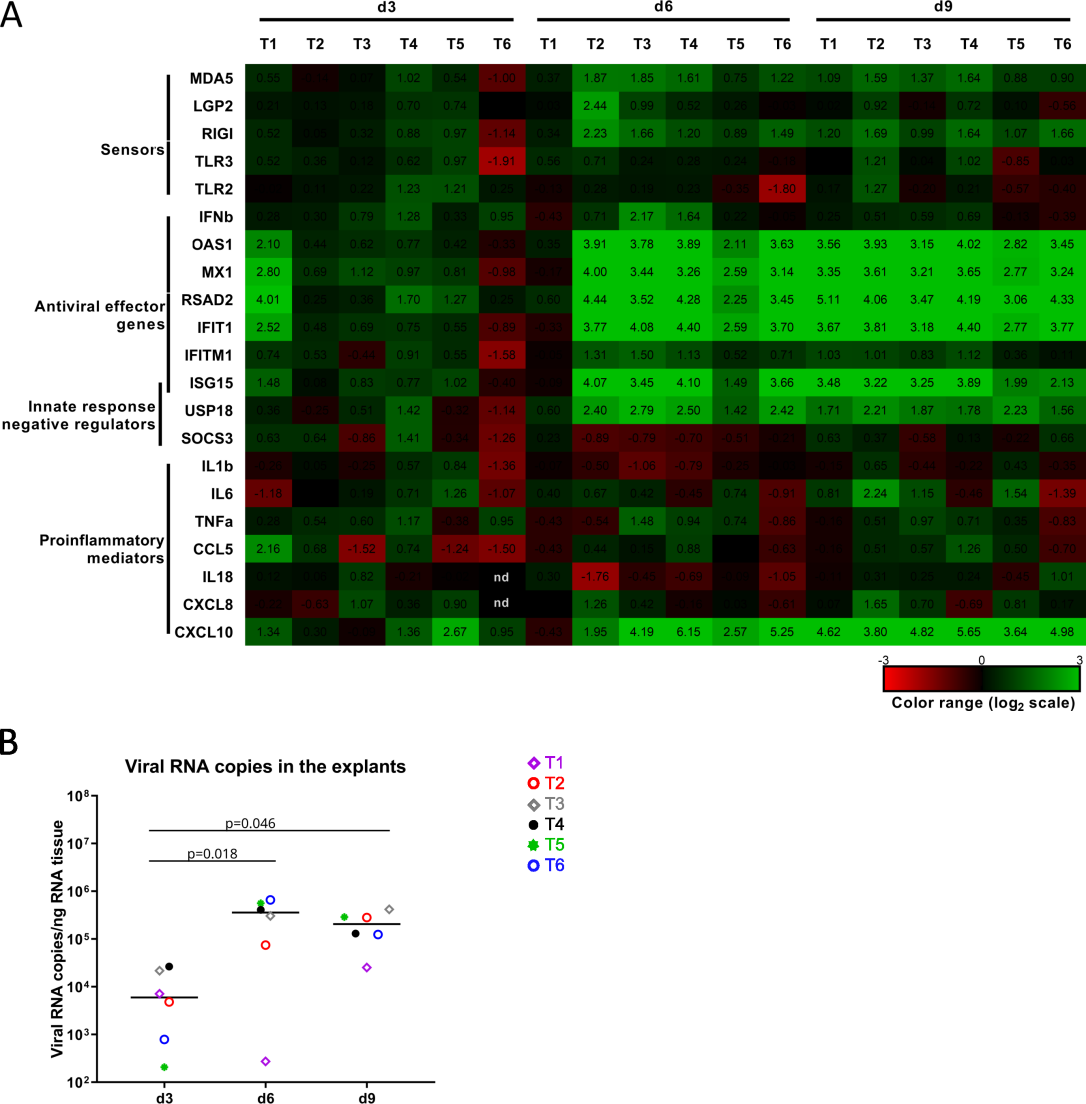
SARS-CoV-2 infection induces antiviral effector genes expression in human testis explants. (**A**) Innate immune gene expression determined by RT-qPCR in testis explants from six donors infected with SARS-CoV-2 beta strain for 3, 6, and 9 dpi (**D3, D6, D9**). Heatmap shows log2-transformed expression ratios between SARS-infected and time-matched, mock-infected controls. Green indicates upregulation, and red, downregulation of mRNA compared with controls. (**B**) Viral loads in the testicular explant tissues used in **A**, detected by RT-qPCR against ORF1b-nsp14 mRNA after total RNA extraction. Mock-infected explants were all negative. Each symbol represents a different donor, and horizontal bars represent median values. Statistical analysis was performed using non-parametric Kruskal-Wallis test followed by Dunn’s multiple-comparison test.

We next investigated the innate immune response of the human testis to SARS-CoV-2 infection *ex vivo* by measuring a panel of innate immune effectors and sensors in RT-qPCR. A range of antiviral effector genes (OAS1, MX1, RSAD2, IFIT1, and ISG15), along with vRNA sensors MDA5 and RIG-I, were consistently increased at 6 and 9 dpi in SARS-CoV-2 beta strain-infected testis explants from all donors (Fig. 3A), except at day 6 for one donor with low vRNA in the explants that time (T1) (Fig. 3B). IFNb and pro-inflammatory cytokines IL1b, IL18, IL-6, TNFa, CCL5, and CXCL18 were not or only weakly increased at all time points in all donors. CXCL10 and USP18, a negative regulator of type I IFN, were upregulated from 6 dpi onwards. In concordance with our previous quantification of vRNA in the testis explant supernatants from these donors (Fig. 1), viral RNA levels within the explant tissues were elevated from 6 dpi onwards (Fig. 3B). Testis explants concomitantly infected by SARS-CoV-2 beta, delta, and omicron BA1 strains showed a weak pro-inflammatory response and an upregulation of antiviral genes in response to the three variants (Fig. S7).

Altogether, our data show that the testis innate response to SARS-CoV-2 infection *ex vivo* is primarily antiviral and that its onsets precede the decline in infectious virus production measured in related supernatants (see Fig. 1B).

### SARS-CoV-2 infection has no major impact on testis morphology or functions *ex vivo*

**Fig 4:**
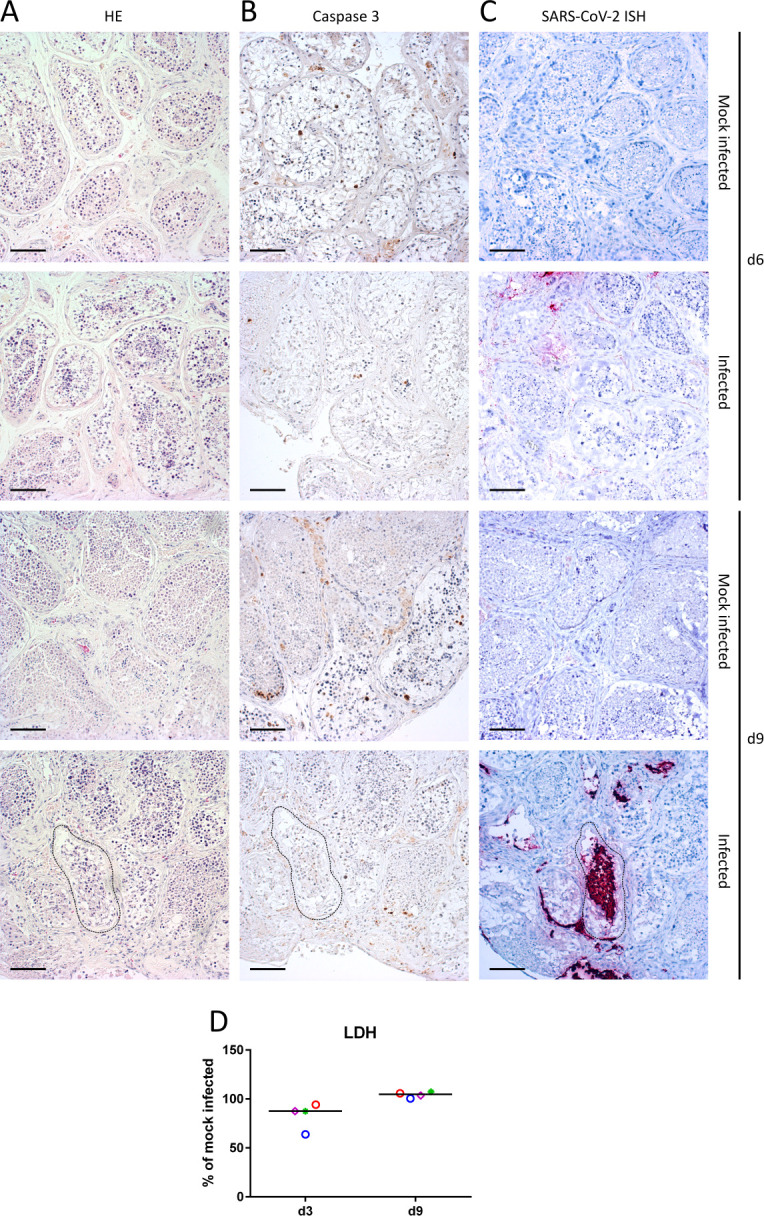
SARS-CoV-2 infection does not alter human testis explant morphology or cell viability *ex vivo.* Representative images of (**A**) hematoxylin-eosin histological staining, (**B**) cleaved caspase-3 apoptosis labeling, and (**C**) RNAScope ISH against SARS-CoV-2 genomic RNA in SARS-CoV-2 beta strain-infected and mock-infected testis explants at 6 and 9 dpi (5-µm serial sections were used for the latter). Scale bars, 100 µm. (**D**) LDH release in testis supernatant from four donors expressed as percentage of mock-infected explants on the corresponding day of culture. Each symbol represents a different donor, and horizontal bars represent median values. No significant difference was found using Mann-Whitney non-parametric test.

**Fig 5:**
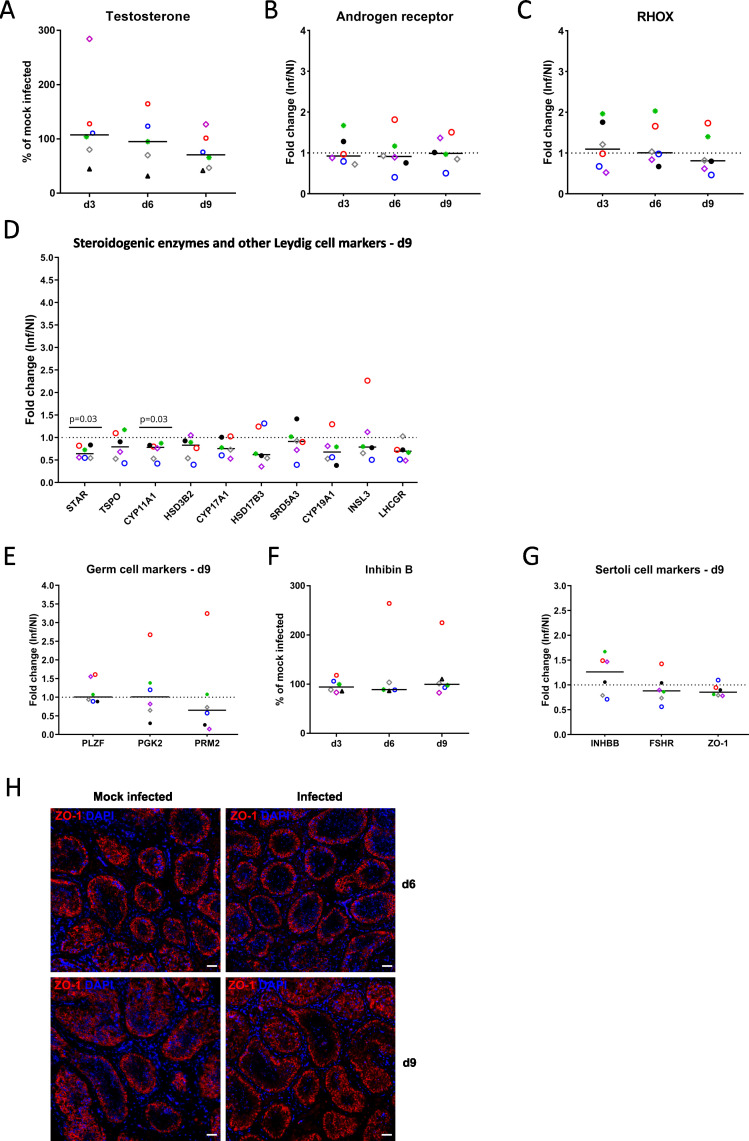
SARS-CoV-2 infection alters Leydig cells steroidogenic enzyme expression *ex vivo.* (**A**) Testosterone release by testis explants from six donors infected with SARS-CoV-2 beta strain, expressed as percentage of mock-infected explants on the corresponding day of culture. No significant difference was found using the non-parametric Kruskal-Wallis test followed by Dunn’s multiple-comparison test. (**B and C**) Fold change in androgen receptor (**B**) and RHOX (**C**) mRNA levels measured by RT-qPCR in testis explants infected by SARS-CoV-2 beta strain versus mock-infected testis up to 9 dpi. No significant difference was found using the one-sample Wilcoxon test. (**D**) Expression profile of steroidogenic enzymes and other Leydig cell markers (INSL3, LHCGR) determined by RT-qPCR at 9 dpi. Values are expressed as fold changes of SARS-CoV-2 beta strain-infected testis (Inf) versus mock-infected testis (NI). Statistical analyses were performed using one-sample Wilcoxon test. (**E**) Expression profile of specific markers for spermatogonia (PLZF), spermatocytes (PGK2), and spermatids (PRM2) at 9 dpi. No significant difference was found using the one-sample Wilcoxon test. (**F**) Inhibin B release by testis explants exposed to SARS-CoV-2 beta strain, expressed as percentage of mock-infected explants on the corresponding day of culture. No significant difference was found using non-parametric Kruskal-Wallis test followed by Dunn’s multiple-comparison test. (**G**) Expression profile of Sertoli cell markers. No significant difference was found using the one-sample Wilcoxon test. (**H**) IHC of Sertoli cell tight junction-associated protein ZO-1 in infected versus non-infected explants at 6 and 9 dpi. Nuclei are stained in blue. Scale bars, 50 µm.

To assess the impact of SARS-CoV-2 in the testis, we first compared tissue morphology through hematoxylin and eosin (H&E) staining, apoptosis through cleaved caspase 3 labeling in IHC, and cell viability through lactate dehydrogenase (LDH) release in supernatants, in SARS-CoV-2 beta strain-infected versus mock-infected testis explants from four to six donors. We did not observe a major impact of the infection on testis architecture (Fig. 4A), apoptosis (Fig. 4B), and overall cell viability (Fig. 4D) over time. We next measured the release of hormones produced by Leydig cells and Sertoli cells in infected versus non-infected testis explants and quantified a range of cell-specific markers in reverse transcripase-quantitative polymerase chain reaction (RT-qPCR) at days 3, 6, and 9 of culture. Overall, testosterone levels were not significantly affected in infected versus uninfected testis supernatants from five to six donors during the culture time frame (Fig. 5A). Androgen receptor and RHOX mRNA expression, which are regulated by testosterone signaling, were not significantly modified in infected explants (Fig. 5B and C). However, testosterone levels showed a tendency to decrease in infected versus non-infected testis explants at day 9 post-infection (*P* = 0,08, Mann-Whitney), with a decrease observed in four out six donors (decrease of testosterone release of 41,85% to 75,61% compared to mock-infected explants from the same donors). The expression of genes responsible for testicular steroidogenesis revealed a concomitant significant decrease of the steroidogenic enzymes STAR and CYP11A1 mRNAs at 9 dpi, along with a global yet non-significant decrease of other Leydig cell markers in infected explants, except for INSL3 at 6 dpi (Fig. 5D; Fig. S8A and B). Germ cell markers (PLZF for spermatogonia, PGK2 for spermatocytes, and PRM2 for spermatids) were not significantly modified by the infection at any time points (Fig. 5E; Fig. S8C and D). The release of inhibin B by Sertoli cells (Fig. 5F) and that of transcripts encoding inhibin B and other Sertoli cell markers (follicle-stimulating hormone (FSH) receptor and ZO-1) were not significantly affected by the infection during the culture time course (Fig. 5G; Fig. S8E and F). ZO-1, a marker of Sertoli cell tight junctions, did not show any significant modification of its distribution upon infection (Fig. 5H). Overall, these data indicate that SARS-CoV-2 has no major detectable impact on the testis tissue during the 9 days of culture, except for a decreased expression of Leydig cell markers toward the end of the culture.

## DISCUSSION

It has been shown that SARS-CoV-2 can spread *in vivo* to extra-pulmonary organs, either through early viremia or infection of the vasculature (2, 4, 5). Despite many speculations since the onset of the Covid-19 pandemic, there has been no formal evidence of SARS-CoV-2 tropism for the human testis. In this study, we demonstrate that SARS-CoV-2 replicates in the human testis *ex vivo* with slow kinetics, first targeting testosterone-producing Leydig cells in the interstitial tissue before infecting Sertoli cells within the seminiferous tubules. The infection appears transient as infectious particles decrease at day 9 after the peak of infection at day 6.

ACE2 protein localized in CYP11+ Leydig cells and vimentin+ Sertoli cells, along with rare DDX4+ germ cells. However, TMPRSS2, the chief protease for Spike protein cleavage and subsequent cell entry, was undetectable, which might contribute to the slow kinetics of testis infection. Other proteases or alternative processes could support SARS-CoV-2 infection of testicular cells. For instance, there is indirect evidence of functional Furin—an alternative protease for SARS-CoV-2 entry (46, 47)—in Leydig cells (48, 49). SARS-CoV-2 entry can also occur in TMPRSS2-negative cells through virion endocytosis and processing of viral spike protein by endolysosomal proteases such as CTSL (46), which are well expressed in Sertoli cells (50). The mechanisms underlying SARS-CoV-2 entry in testicular cells and its slow replication kinetics within the explants will require further investigation.

Interestingly, the decreased release of infectious virions by testis explants at day 9 contrasts with our previous findings of sustained infectious ZIKV production in testis explants and germ cells (36), an established reservoir for ZIKV lacking antiviral responses (37, 40). It suggests that unlike with Zika virus, the human testis may only harbor replicative SARS-CoV-2 for short duration. This could be due to several non-exclusive mechanisms, including (i) cell death or metabolic dysfunction of the main virions producing cell population. While no increased cell mortality was observed in infected explants, the trend for decreased expression of Leydig cell markers at 9 dpi suggests a negative impact of SARS-CoV-2 infection on their metabolism, which could affect their ability to support full viral replication; (ii) control of viral replication by the cellular antiviral response. Indeed, SARS-CoV-2 is remarkably sensitive to cellular antiviral responses *in vitro*, even once infection is established (51, 52). Cellular antiviral responses mediated by type I IFN are also crucial for the successful control of SARS-CoV-2 infection in patients (53). OAS1 and Mx1, which are strong inhibitors of SARS-CoV-2 replication in patients (54), were upregulated in SARS-CoV-2-infected testis explants at peak replication and their induction preceded the decline in virus titer. ISG induction likely occurred upon recognition of virus material by sensing receptors such as MDA5 and RIG-I (which mRNAs were slightly upregulated by SARS-CoV-2 infection) in an IFN-independent process, as described in other models (54); (iii) the viral titer decrease at day 9 could also result from the inability to produce mature virions by the principal cell types infected at this time point, i.e., Sertoli cells. The drop of infectious titer at day 9, while vRNA levels are sustained in supernatants and primarily localized within the seminiferous tubules, suggests abortive infection in Sertoli cells. In support of this hypothesis, primary commercial human Sertoli cells failed to produce significant quantities of infectious SARS-CoV-2 *in vitro (* 29, 32); (iv) the low level of replication of SARS-CoV-2 in the testis might be insufficient to sustain infection of the tissue beyond the first few days post-exposure.

Consistent with our results *ex vivo*, several studies in animal models and men suggest that SARS-CoV-2 transiently infects the testis to low levels *in vivo*. In men, no sign of recent SARS-CoV-2 replication was evidenced in the testes beyond 14 days post-infection (4). In a model of Syrian hamsters, SARS-CoV-2 was detected in testes up to 4 dpi by RT-qPCR but resolved thereafter in most animals (32). In transgenic mice, the infection of testicular interstitial tissue, which occurred prior to that of seminiferous tubules as in our *ex vivo* model, was accompanied by immune infiltrates and cleared after a few days (31). In patients, the inconsistent detection of viral material in testes at autopsy (2, 4, 13, 23, 25 – 27) and of vRNA shedding in semen (15, 16, 19, 24) suggests that SARS-CoV-2 does not replicate for extended duration in the male genital tract. Although isolated cases of viral persistence in the male genital tract may occur, as reported in one patient up to 81 days post-symptoms (24), our data are in favor of transient infection of the testis by SARS-CoV-2. A study mixing 2D and 3D culture systems of primary commercial human Leydig and Sertoli cells with digested seminiferous tubules failed to detect productive infection of testicular cells by SARS-CoV-2 (29). Distinct results were obtained by Li et al. who showed SARS-CoV-2 entry into human Leydig-like cells derived from hiPSC lines (that unlike their commercial counterparts expressed the Leydig cell marker 3b-HSD and produced testosterone) and evidenced SARS-CoV-2 nucleocapsid in hamsters’ Leydig cells *in vivo* (55). Unlike our model based on fresh whole human testes set immediately post-mortem into thoroughly characterized organotypic culture (56), these studies (29) used poorly characterized isolated testicular cells of unclear origin and reconstituted testicular 2D and 3D culture systems from dissociated cryopreserved testis fragments that cannot recapitulate the complex testis architecture and cellular interactions, previously showed to play a key role for viral infection in the testis (36, 57). The relevance to viral tropism *in vivo* of our model of human testis has been largely validated in human cohorts and animal models for various pathogens (40, 58 – 60). The low frequency of seminal shedding reported in SARS-CoV-2-infected men (61) is in agreement with our findings. Indeed, virions produced by interstitial Leydig cells are naturally segregated from the seminal compartment *in vivo* by the blood testis barrier, and Sertoli cells appear to be poor producers of infectious viral particles, thus limiting the release of virions in the seminal compartment and semen.

Pro-inflammatory response was minimal in SARS-CoV-2-infected testis explants, with only CXCL10 upregulation, along with marginal IFNb induction. TLR2, which signaling upon sensing of SARS-CoV-2 envelope protein is required for inflammatory cytokines release in patients (62) and *in vitro* (29), was not upregulated in testis explants. This contrasts with highly permissive lung and gut models, in which SARS-CoV-2 induced an early antiviral response and a broad pro-inflammatory response (9, 63). While this restricted immune response might reflect the limited infection of the testis by SARS-CoV-2, it is important to note that it is similar to the innate response we previously reported upon Zika virus infection (36) or Poly(I:C) exposure (37). We hypothesize that in the immune-suppressed testis environment, the induction of innate immune responses is tightly controlled in order to preserve the organ homeostasis, especially that of pro-inflammatory cytokines and IFNb, which over-expression can lead to sterility (38, 64). The mechanisms that regulate innate immune responses in the human testis tissue are currently under study in our laboratory.

The overall testis tissue morphology and viability appeared unmodified by SARS-CoV-2 infection during the 9-day culture. This could be due to the slow replication kinetics of SARS-CoV-2 in this tissue (peak at a median of 4,15 log10 PFU/mL at day 6 of culture) and is in contrast to the more permissive *ex vivo* models of lungs and gut (over 4 log10 PFU/mL within 24 h to 72 h of culture) that show hallmarks of cell death (9, 65). In the testes from deceased patients who suffered from severe COVID-19 and in animal models, severe morphological damages associated with immune cell infiltrates have been reported (13, 23, 66), although SARS-CoV-2 nucleic acids or proteins were only occasionally detected (2, 13, 23, 25, 33, 35). These damages are typical of the consequences of testis inflammation (38) upon either viral replication in the testis (e.g., mumps virus) or infection with non-testis tropic viruses that induce elevated and prolonged systemic inflammation, such as influenza virus (38). Our data showing low level of viral replication and lack of pro-inflammatory response in the testis tissue suggest that the testicular inflammation and damages observed *in vivo* result from indirect effects of the infection onto the testis, such as elevated systemic inflammation inducing immune infiltrates and cytotoxicity in testicular cells (29), combined with medication in severely ill patients, rather than from infection of the testis itself. Nevertheless, the intra-testicular injection of SARS-CoV-2 in transgenic mice expressing human ACE2 led to massive T cell infiltration and seminiferous tubule disruption, indicating that testicular infection itself—and not systemic inflammation— can induce testis inflammation in rodents (31). However, testis innate responses to viral stimuli drastically differ between human and rodent testis (37, 38), and therefore, infection outcomes in men cannot be extrapolated from animal models.

Our testis model has been extensively used to determine the impact of various biological and chemical agents on testicular morphology and functions, including alterations of testosterone secretion (36, 41, 56, 58, 67 – 69). Nevertheless, this model has several limitations regarding testicular pathogenesis: first, we cannot assess the deleterious effect of systemic inflammation and immune cells infiltrating the testis. Second, we cannot rule out that the relatively short duration of the testis culture (9 days) and the focal nature of the infection may have precluded the detection of delayed or localized effects of SARS-CoV-2 on testis morphology and functions. For instance, scar from transient replication in Leydig cells might contribute to testis damages and hormonal alterations *in vivo*. Thus, a significant decrease in Leydig cells’ steroidogenic enzymes was observed toward the end of the culture of human testis explants, suggesting a direct effect of SARS-CoV-2 onto this infected cell type. In line, elevated luteinizing hormone (LH) concentrations associated with low to normal testosterone levels indicating primary testicular failure were reported in a subset of Covid-19 patients (19, 21, 70). Testosterone levels in men suffering from Covid-19 were lower than that in critically ill men without Covid-19 (21), suggesting an impact of SARS-CoV-2 on Leydig cells. A proteomic analysis showed that the few dysregulated proteins in the testis from deceased patients with Covid-19 were primarily of Leydig cells’ origins and essentially involved in cholesterol bio-synthesis (3). Moreover, plasmatic concentrations of insulin-like factor 3, a Leydig cell product for which a transient decrease was measured in infected testis explants, were diminished during acute phase in patients (70). In contrast, inhibin B—a marker of Sertoli cell function—and its regulatory gonadotropin FSH were unchanged in most patients with moderate to severe Covid-19 (20, 71). In agreement, Sertoli cell markers including inhibin B release were unaffected in infected testis explants. Altogether, these data suggest that SARS-CoV-2 could directly affect Leydig cells through yet unknown mechanisms. ACE2 perturbation upon SARS CoV-2 binding could be involved, as components of the renin-angiotensin system expressed by Leydig cells are suspected to modulate testosterone production (72 – 74). The downregulation of ACE2 in Leydig cells has also been correlated with spermatogenesis impairment in men (75). Therefore, beside fever and medications (22, 38), SARS-CoV-2 infection of Leydig cells might be involved in the alterations of sperm parameters reported in patients with Covid-19 (14, 19), a subset of which only had mild symptoms and no fever (17).

In conclusion, this study demonstrates for the first time that SARS-CoV-2 replicates with slow kinetics in the human testis *ex vivo*. The infection had no major impact on testis morphology and functions during the culture time frame. Overall, these data suggest that the dramatic damages reported in the testis from deceased patients are unlikely to result from a direct effect of SARS-CoV-2 in the testis. Although the infection appeared transient, the long-term impact of SARS-CoV-2 infection on Leydig cell functions and spermatogenesis requires further investigations, as a decrease in Leydig cells’ steroidogenic enzymes was observed at the end of the 9-day culture period. In that respect, the follow-up of semen parameters and hormonal levels in infected men with mild symptoms (i.e., without high inflammation and fever or medication) will be important.

## MATERIALS AND METHODS

### Virus

SARS-CoV-2 beta strain was isolated during the 2020 outbreak in France and passaged three times in Vero cells (BetaCoV/France/IDF0372/2020, European Virus Archive). SARS-CoV-2 delta and omicron BA-1 strains have been described previously (76, 77). VeroE6 cells were maintained as already described (36). To produce viral stocks, VeroE6 cells were infected at a multiplicity of infection (MOI) of 10^−4^ in serum-free medium with 1 µg/mL TPCK-trypsin (Sigma-Aldrich) for 2 h and cultured for 3 days in complete medium at a final serum concentration of 5%. Supernatants were then harvested, centrifuged, filtered (0,45 µm), aliquoted, and frozen at −80°C.

### Organotypic culture of human testis explants and infection

Human testis samples, obtained from deceased (*n* = 7) or prostate cancer patients (*n* = 1, T7) at Rennes University Hospital, were dissected into 3-mm^3^ explants and cultured as we previously described (36). For infection, explants (four sections per well) were transferred in 500 µL of serum-free culture medium in the presence or absence of 7 × 10^4^ PFU SARS-CoV-2 (corresponding to 29 × 10^8^ vRNA copies for the beta strain and to 4,2 × 10^9^ and 9,9 × 10^9^ for delta and omicron BA-1, respectively). After an overnight incubation, explants were washed three times in PBS, transferred onto polyethylene terephthalate insert (3-µm high-density pores) in 12-well plates (two explants per well) containing 1 mL of culture medium. Six hours later, the medium was changed again to further wash away potential residual virus input (time 0 for sample collection). To determine replication capacity, we measured viral infectious particles and viral RNA at 6 hpi, which was set as the baseline for assessing residual inoculated virus after incubation and washes. The release of viral RNA and infectious virions was then measured on days 3, 6, and 9. For each experimental condition, three wells were tested, and explants were cultured and processed as we previously described (36).

### Isolation and infection of testicular germ cells

Primary testicular cells were prepared as we previously described (36). Upon isolation and overnight culture, non-adherent germ cells were collected and incubated in the presence or absence of SARS-COV-2 beta strain at an MOI of 1 (i.e., 10^6^ PFU/million cells) for 2 h at 37°C with 5% CO_2_. Cells were trypsinized for 5 min at 37°C before inactivation and then cultured onto six-well plates at a density of 1 million cells per milliliter in supplemented StemPro-34 (Invitrogen).

### RT-qPCR

Total RNA was extracted using QIAmp vRNA (for supernatants) and RNAeasy isolation kit (for tissues and cells) and treated with DNase (all from Qiagen). RNA extracted from culture supernatants was subjected to RT-qPCR using GoTaq Probe 1-Step RT-qPCR System (Promega) and SARS-CoV-2 primers and probes as follows: HKU-ORF1b-nsp14 forward TGGGGTTTTACAGGTAACCT, reverse AACACGCTTAACAAAGCACTC, and probe TAGTTGTGATGCAATCATGACTAG (78). A full-length viral genome RNA (EVA, isolate SARS-CoV-2/human/ITA/INMI1/2020) was used as standard, and serial dilutions of a known number of copies of vRNA were systematically run. Primers for the relative quantification of steroidogenesis enzymes mRNA, testicular cell markers mRNA, and innate immune response effector genes mRNA are listed in the Table S1 and were either used previously (36) or designed using the Primer-BLAST tool. Total RNA extracted from tissues and cells was reverse transcribed using the iScript cDNA Synthesis Kit and then subjected to RT-qPCR using the iTAq SYBR green mix (all from Bio-Rad) as previously described (36).

### Quantification of infectious viral titer

Infectious viruses were quantified by plaque assay as described (79). Briefly, 2,5 × 10^5^ VeroE6 cells were seeded in 12-well plates and allowed to grow to confluence for 24 h. Medium was then removed, and cells were incubated with 125 µL of serial dilutions of culture supernatants for 1 h at 37°C. Cells were overlaid with 0,8 mL of a 1:1 mixture of DMEM 2× with 4% SVF and 2% methylcellulose in ddH20 and incubated at 37°C for 4 days. The overlay media were washed away, and cells were fixed in 4% paraformaldehyde for 1 h before staining with a 1% (wt/vol) crystal violet solution in 20% ethanol. The number of plaques was then counted, and virus titer was defined as the number of plaque-forming units per milliliter.

### RNAscope ISH, IHC, and histology assessment

Testis explants were fixed in 4% formaldehyde and embedded in paraffin. RNAscope ISH and dual fluorescence RNAscope ISH-IHC were performed as previously described (36). For RNAscope, antisense probes (nucleotide 21,563–25,384, catalog 848561) and sense probes (nucleotide 21,631–23,303, catalog 845701) targeting the viral spike encoding sequence were obtained from Advanced Cell Diagnostics. For dual fluorescence RNAScope ISH-IHC or single IHC, antibodies and specific conditions are specified in the Table S2. Sections of mock-infected testis explants or sections stained with isotype antibody control were systematically used as negative control. Colorimetric stained sections were examined and photographed under a light microscope (Olympus BX51) or captured with a scanner NanoZoomer 2.0 RS (Hamamatsu, Tokyo, Japan) at 40× magnification. Fluorescent images were acquired with the Zeiss Axio Imager system connected to Zen software or with the SP8 confocal system (Leica) connected to LAS software and analyzed using Fiji software.

### Bioinformatic analysis of RNA sequencing data

Adult human testis bulk RNA sequencing (42 – 45) data sets were obtained from the ReproGenomic Viewer website (80) and were plotted using average FPKM values of all individual samples for each data set.

### Tissue viability assay

Global tissue viability was assessed by measuring the LDH release in culture medium using the enzymatic fluorometric assay CytoTox-ONE Homogeneous Integrity Assay (Promega) according to the manufacturer’s instructions.

### Hormone quantification

Testosterone (T) was measured using an in-house liquid chromatography-mass spectrometry after liquid extraction using Agilent’s 1290 Infinity II LC System and MSMS 6495. Mean intra-assay coefficients of variation were 3.7% and 3.3% for T concentrations of 0.080 and 0.274 nmol/L, respectively; inter-assay coefficients of variation were 4.71% and 5.98% for T concentrations of 2.254 and 8.934 nmol/L, respectively. Inhibin B concentration was measured using Beckman Coulter’s Gen II ELISA Kit (DSL-10684100) according to the manufacturer’s instructions. The limit of quantification was 5 ng/L.

### Immunocytofluorescence

Testicular germ cells put onto polylysine-coated glass coverslips were fixed in 4% paraformaldehyde for 20 min at room temperature. Immunocytofluorescence was performed as previously described (36). Viral material was detected with antibodies against SARS-CoV-2 nucleoprotein (1 µg/mL, GeneTex, 3851) or viral double-strand dsRNA (1 µg/mL, anti-dsRNA J2, Scicons). Infected cell characterization was performed using rabbit anti-DDX4 (5 µg/mL, Abcam, ab13840). Isotype control antibodies or non-infected cells were used as negative controls. Images were acquired with the SP8 confocal system (Leica) connected to LAS software and analyzed using Fiji software.

### Statistics

When comparing independent sets of samples, we used unpaired Mann-Whitney tests (two sets to compare) or Kruskal-Wallis tests followed by Dunn tests (more than two sets to compare). When repeated measurements along time data were paired, Wilcoxon signed-rank test (for two repetitions) or Friedman test followed by Dunn test (more than two repetitions) were implemented. Data expressed as fold change (ratio between infected and their respective control) were compared to 1 using one-sample Wilcoxon test. Patient hormonal levels, when outside of the normal range, were compared to the minimal reference values using one-sample Wilcoxon test. Statistical significance threshold was set at 0.05. All statistical analyzes were performed using GraphPad Prism 6 software and are specified in the figure legends.

### Study approval

Normal testes were obtained at autopsy of organ donors or for one patient (T7) after orchiectomy and processed within 2 h of surgery. The procedure was approved by Ethics Committee Ouest V, Rennes, France (authorization DC-2016-2783), and the French National Agency for Bio-medical Research (authorization PF S09-015).

## SUPPLEMENTAL MATERIAL

Legends of supplemental figures jvi.01104-23-s0001.docx Legends of Fig. S1 to S8.

Supplemental figures and tables jvi.01104-23-s0002.pdf Fig. S1 to S8 and Tables S1 and S2.

ASM does not own the copyrights to Supplemental Material that may be linked to, or accessed through, an article. The authors have granted ASM a non-exclusive, world-wide license to publish the Supplemental Material files. Please contact the corresponding author directly for reuse.
