# Supplementary material for: SARS-CoV-2 replicates in the human testis with slow kinetics and has no major deleterious effects ex vivo
Source: J Virol. 2023 Oct 13;97(10):e01104-23. doi: 10.1128/jvi.01104-23 (PMC10653996; doi:10.1128/jvi.01104-23)
Supplement: Legends of supplemental figures — Legends of Fig. S1 to S8. [file jvi.01104-23-s0001.docx]

**Supplemental Fig 1. Identification of internalized SARS-CoV-2 replicative RNA in human testicular cells**

RNAscope ISH using probes targeting SARS-CoV-2 replicative (R) RNA was coupled with immunofluorescence for cell markers to label either (A) Cyp11A1+ Leydig cells, (B) αSMA+ peritubular cells, (C) Vimentin+ Sertoli cells, in SARS-CoV-2 Beta strain infected explants. Confocal z-stack maximum projections (left panels) and z-stack along with cross-sectional xz orthogonal viewing from the indicated positions (a, b, c, right panels) are presented to visualize internalized viral RNA (arrowhead). Nuclei are stained in blue. Scale bars: 20µm.

**Supplemental Fig 2. *In situ* characterization of infected cells in testis explants infected with SARS-CoV-2 Delta and Omicron BA-1 strains**

**(A-H)** IHC against SARS-CoV-2 nucleoprotein (NP) was coupled with antibodies specific to label Cyp17A1+ Leydig cells **(A, D)**, αSMA+ peritubular cells **(B, E),** vimentin+ Sertoli cells **(C, F)** in testis explants infected by SARS-CoV-2 Delta **(A-C)** or Omicron BA-1 **(D-F)** strains. No staining for SARS-CoV-2 and for cell markers isotypes were ever observed in mock-infected testis **(G, H)**. Nuclei are stained in blue. Scale bars: 20µm.

**Supplemental Fig 3. SARS-CoV-2 infection of isolated testicular germ cells**

Primary testicular germ cells (TGC) isolated from seminiferous tubules were infected 2 hours with SARS-CoV-2 Beta strain (MOI of 1, i.e. 10^6^ PFU per million cells), and cultured for 4 days as we previously described ^35^. **(A)** Viral titers were measured by infectivity assay on VeroE6 cells. The 2 donors tested are represented by a distinct symbol. The dotted line indicates the detection limit. **(B)** SARS-CoV-2 replicative RNA was localized in germ cells by IHC using an antibody targeting double-stranded RNA (dsRNA), an intermediary of SARS-CoV-2 replication coupled with an antibody against the germ cell marker DDX4. Nuclei are stained in blue. Scale bars: 20µm.

**Supplemental Fig 4. Expression of putative entry factors for SARS-CoV-2 infection in the human testis**

**(A)** Expression levels of known entry factors for SARS-CoV-2 in the testis. Showed are the mRNA expression levels from publicly available bulk RNA sequencing data, represented as Fragment Per Kilobase Million (FPKM) values. Each dot represents the mean FPKM value obtained for testis samples from different donors (n=1 to 8) in each of the four studies ^41–44^ (testis from 13 donors in total). Horizontal bars represent median values. **(B)** Relative expression levels of entry factors for SARS-CoV-2 in the testis. mRNA expression was quantified by RT-qPCR and is presented as the ratio of cycle threshold (Ct) values for each entry factors versus actin in testis explants. Each symbol represents a non-infected testis explant from a distinct donor. Horizontal bars represent median values. Transparent symbols represent samples with a raw Ct value below 29 for the entry factor.

**Supplemental Fig 5. ACE2 is expressed by Leydig and Sertoli cells in the human testis.**

**(A-G)** Representative images of immunofluorescent labelling of ACE2 and cell markers in non-infected testis explants (n=4 independent donors). **(B)** No ACE2 colocalization was observed with CD68/CD163+ macrophages**. (A)** ACE2 was expressed by Cyp11A1+ Leydig cells localized in the interstitial tissue and bordering the seminiferous tubules (white arrow head)**. (C)** SMA+ peritubular cells were negative for ACE2**. (D-F)** In the seminiferous tubules, ACE2 was expressed by Vimentin+ Sertoli cells **(D)**, whereas DDX4+ germ cells were overall negative **(E),** except from the rare ACE2/DDX4 co-labelling detected in isolated germ cells within a few seminiferous tubules (white arrow) **(F).** Nuclei are stained in blue. **(G)** Goat and rabbit IgG isotypes are shown as representative negative controls**.** Scale bars: 20µm. **(H)** Fold change in ACE2 mRNA levels were measured by RT-qPCR in testis explants infected by SARS-CoV-2 (Inf) versus mock infected (NI) testis along the 9-day culture period. Each symbol represents a different donor. Bars represent median values. No significant difference was found using the One Sample Wilcoxon test.

**Supplemental Fig 6. ACE2 expression during culture of infected and mock-infected testis explants and absence of detection of TMPRSS2 protein in the testis**

**(A)** Relative expression levels of ACE2 mRNA in the mock-infected (NI) and infected (Inf) testis explants at day 3 (d3), day 6 (d6) and day 9 (d9) of culture. mRNA expression was quantified by RT-qPCR and is presented as the ratio of cycle threshold (Ct) values for ACE2 versus actin in testis explants. Each dot represents a different donor and horizontal bars represent median values. No significant difference was found using Mann Whitney test. **(B)** Representative images of immunofluorescent labelling of ACE2 in mock-infected and infected testis explants at day 0 (d0), day 3 (d3) and day 9 (d9) of culture. No changes in intensity or localization of the staining was observed over the culture and infection course. No staining for ACE2 isotypes (gIgG) was ever observed in infected or mock-infected testis. **(C)** Representative images of immunofluorescent labelling of TMPRSS2 in non-infected prostate and testis. TMPRSS2 staining was found in epithelial cells of the prostate, whereas no staining was observed for TMPRSS2 in testis explants. Staining specificity in the prostate was controlled using isotype (rIgG). Nuclei are stained in blue. Scale bars: 50µm.

**Supplemental Fig 7. Innate immune response in testis explants exposed to SARS-CoV-2 Beta, Delta and Omicron BA-1 strains**

**(A)** Innate immune gene expression determined by RT-qPCR in testis explants from one testis donor infected with SARS-CoV-2 Beta (B), Delta (D) or Omicron BA-1 (O) strains for 3, 6 and 9 dpi (d3, d6, d9). Heatmap shows log2-transformed expression ratios between SARS-infected and time-matched mock-infected controls. Green indicates upregulation and red downregulation of mRNA compared with controls. **(B)** Viral loads in the testicular explants tissues used in (A), detected by RT-qPCR against ORF1b-nsp14 mRNA after total RNA extraction. Mock-infected explants were all negative. Each strain is represented by a specific symbol.

**Supplemental Fig 8. Testicular cell markers expression in testis explants infected by SARS-CoV-2**

Expression profile of transcripts encoding **(A, B)** steroidogenic enzymes and other Leydig cell markers (INSL3, LHCGR); **(C, D)** markers of spermatogonia (PLZF), spermatocytes (PGK2) and spermatids (PRM2); **(E, F)** markers of Sertoli cells, as determined by RT-qPCR at 3 **(A, C, E)** and 6 dpi **(B, D, F)**. Values are expressed as fold changes of SARS-CoV-2 Beta strain infected testis (Inf) versus mock-infected testis (NI). Each symbol represents a different donor and horizontal bars represent median values. Statistical analyses were performed using One Sample Wilcoxon test.
