# Supplementary material for: SARS-CoV-2 replicates in the human testis with slow kinetics and has no major deleterious effects ex vivo
Source: J Virol. 2023 Oct 13;97(10):e01104-23. doi: 10.1128/jvi.01104-23 (PMC10653996; doi:10.1128/jvi.01104-23)
Supplement: Supplemental figures and tables — Fig. S1 to S8 and Tables S1 and S2. [file jvi.01104-23-s0002.pdf]

Suppl. Figure 1

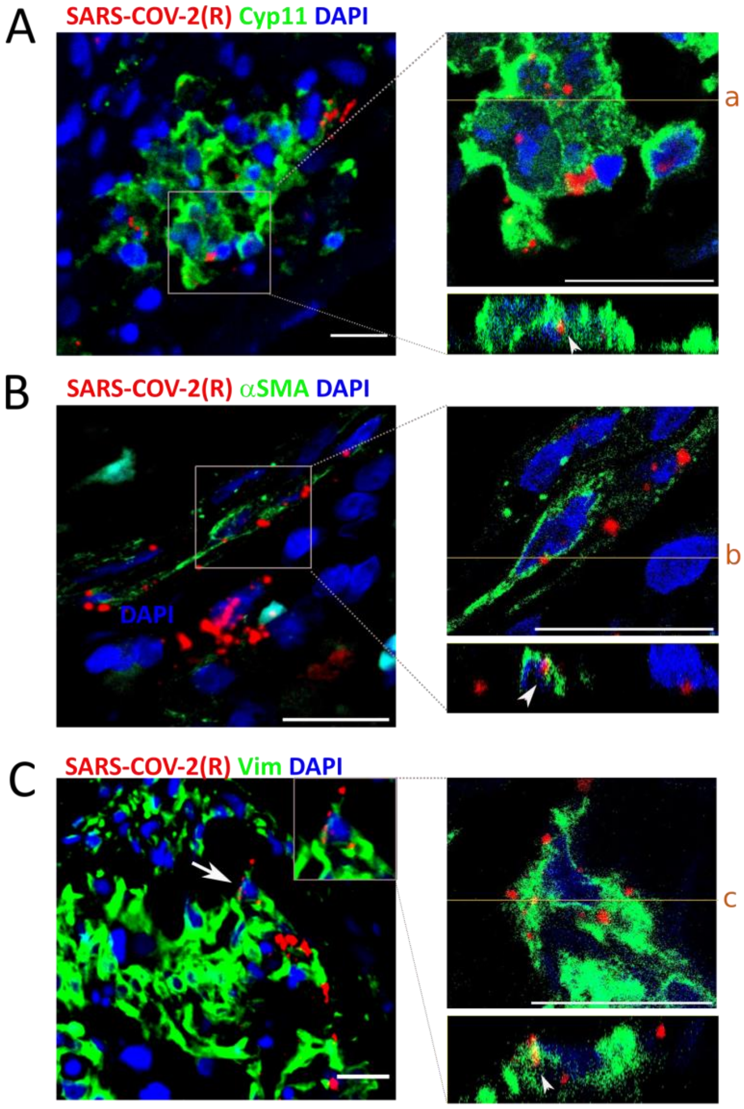

Suppl. Figure 2

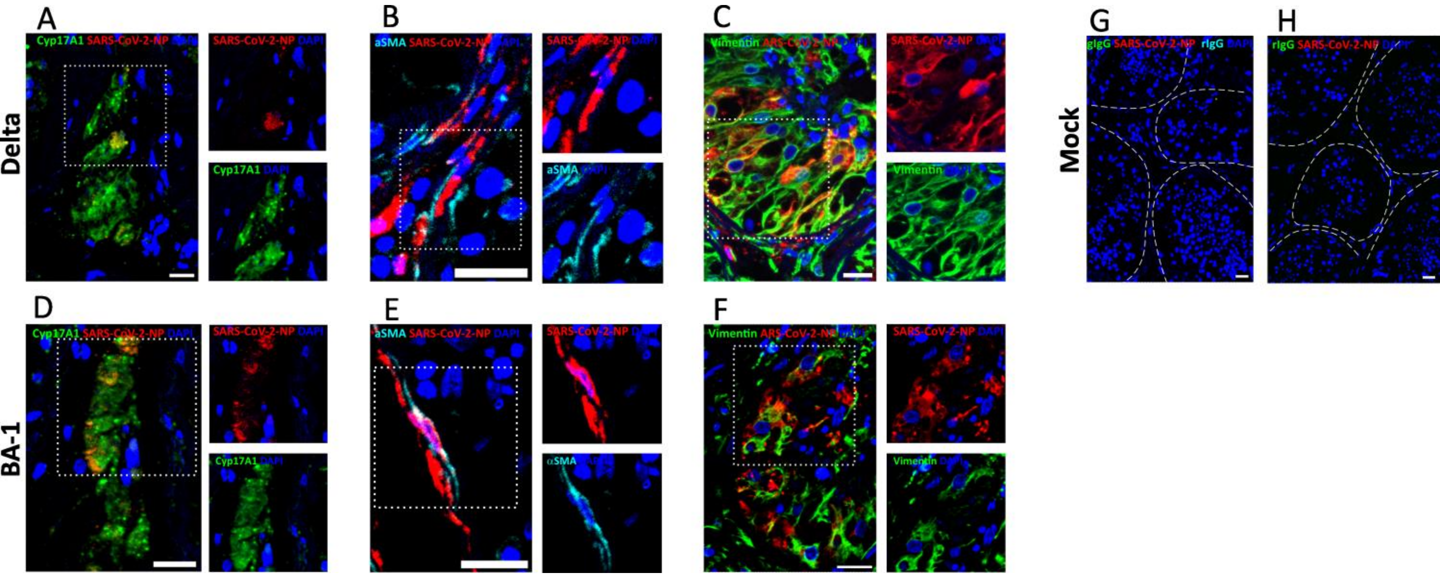

Suppl. Figure 3

A

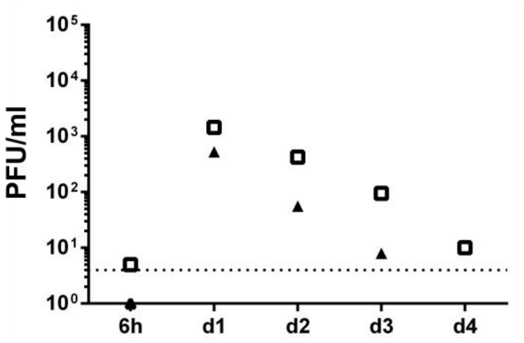

B

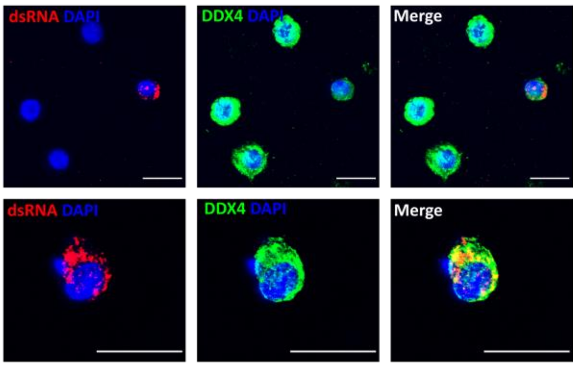

Suppl. Figure 4

A

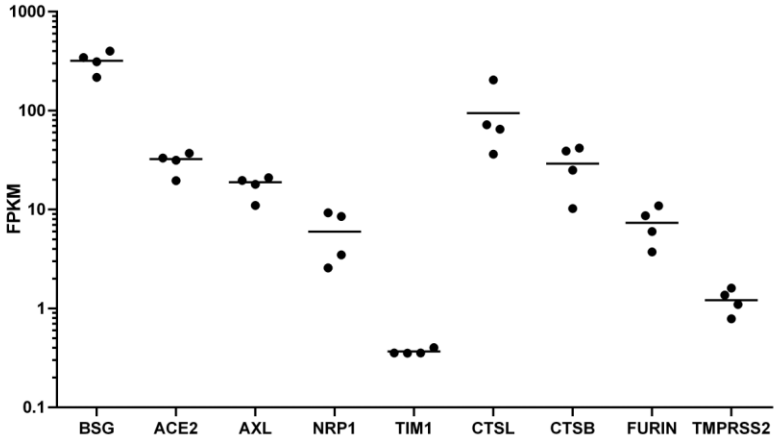

B

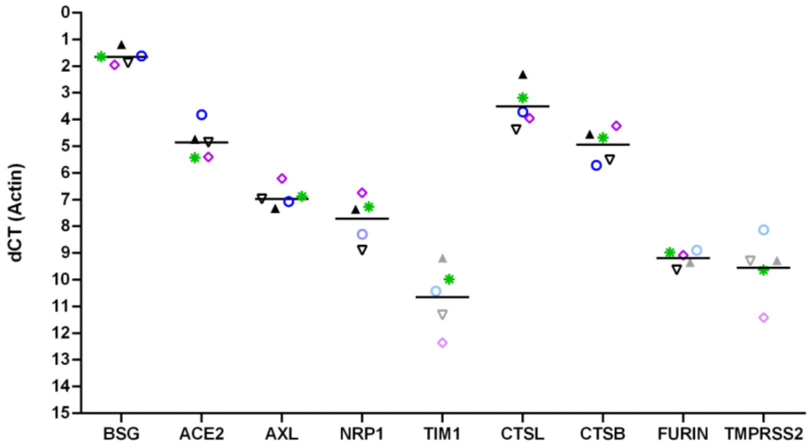

Suppl. Figure 5

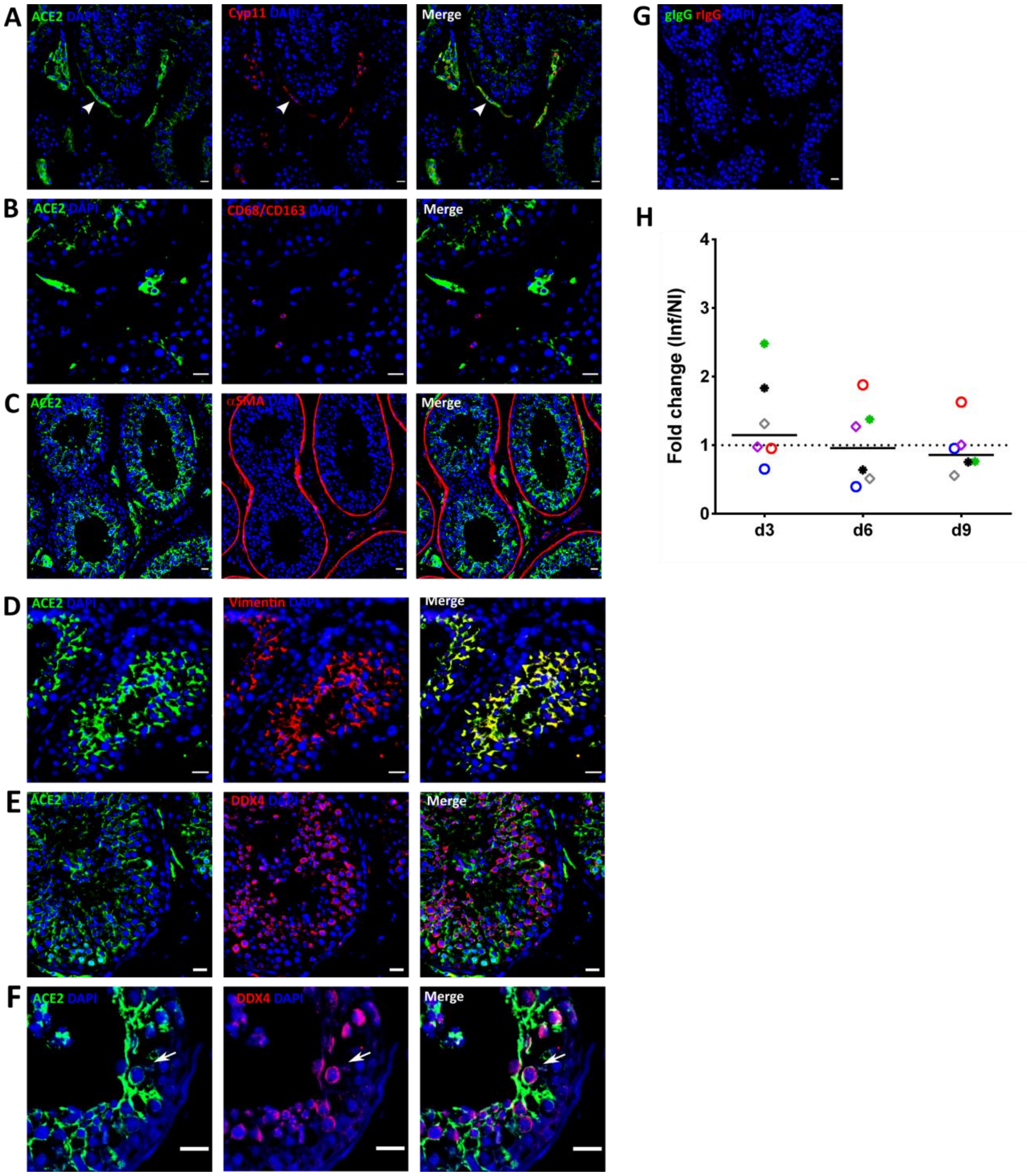

Suppl. Figure 6

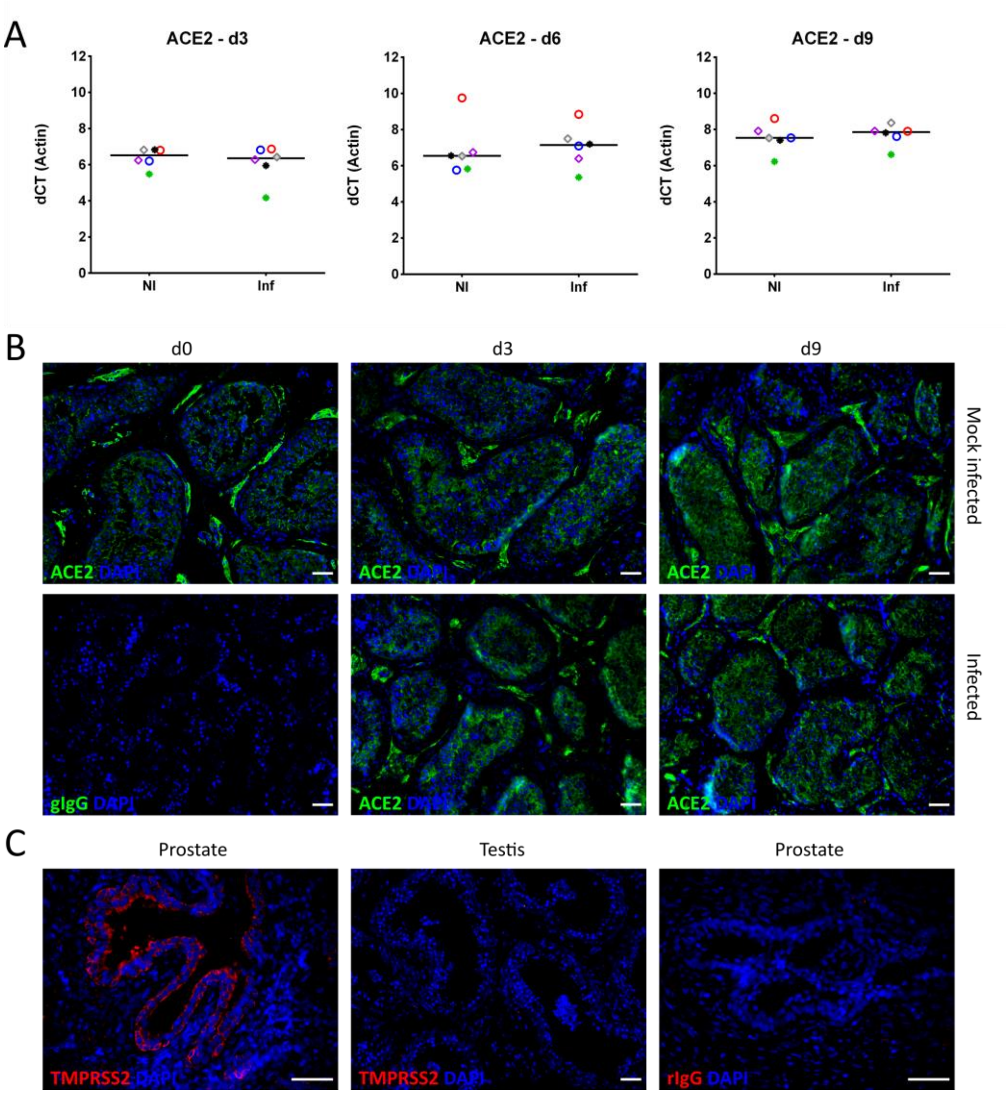

Suppl. Figure 7

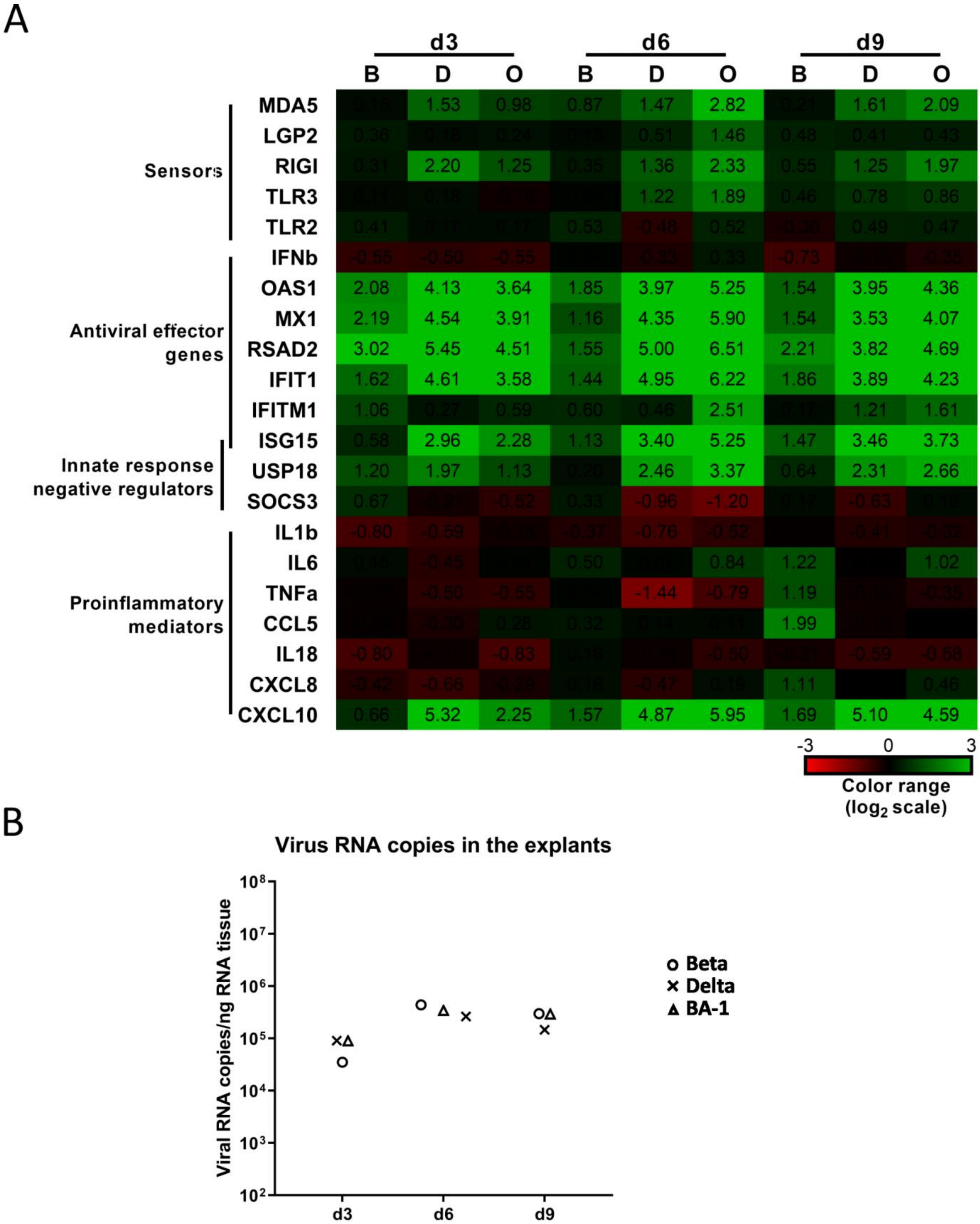

Suppl. Figure 8

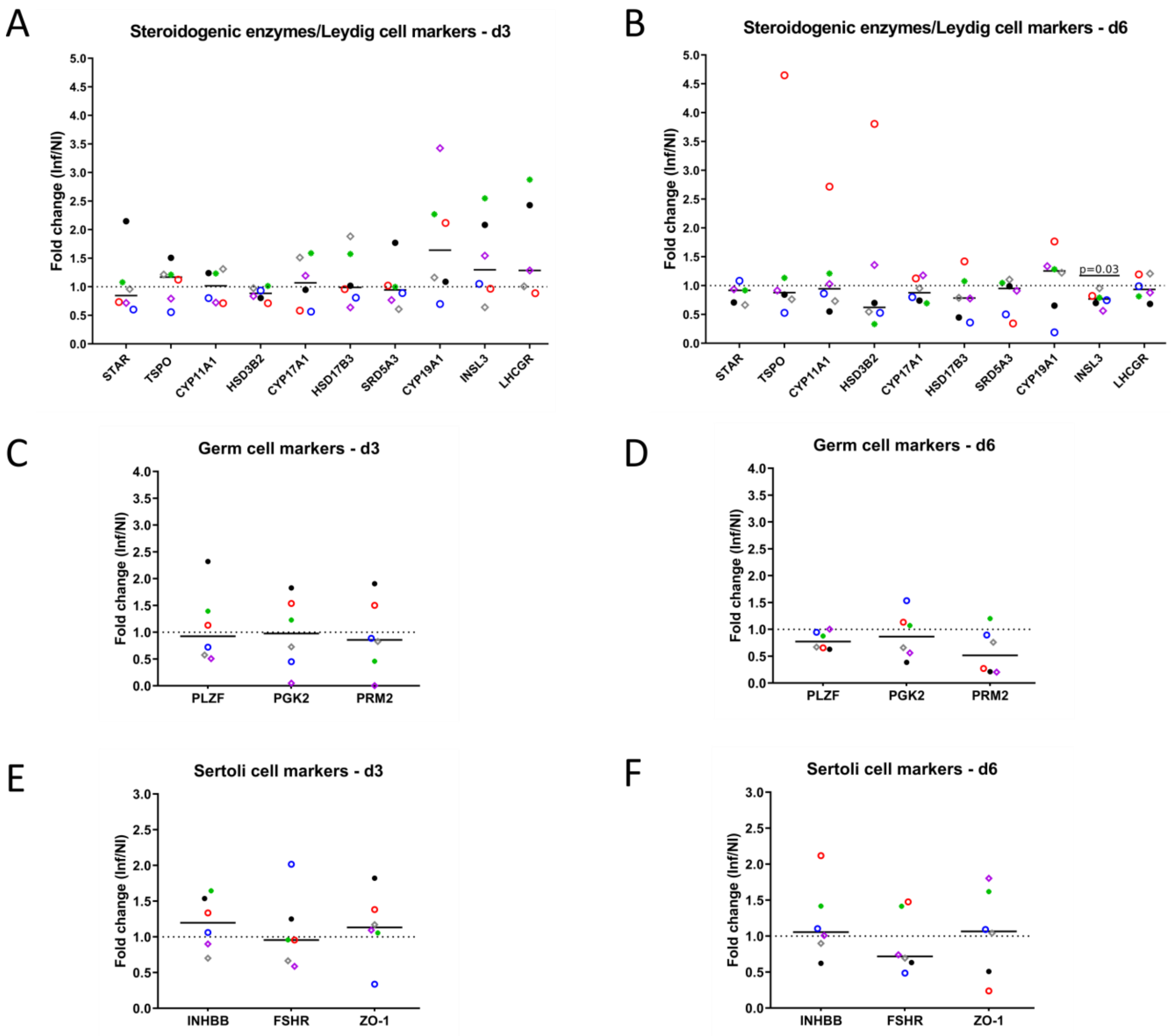

Suppl Table 1

Supplementary Table 1. Primers used for RT-qPCR.

Gene	Forward primer	Reverse primer
ACE2	GTGGTGGGAGATGAAGCGAGAG	AGAGATGCGGGGTCACAGTATG
ACTB	TACAGCTTCACCACCACGG	TGCTCGAAGTCCAGGGCGA
AR	TACGGGGACATGCGTTTGGAG	ACAGATCAGGCAGGTCTTCTGG
AXL	TGGTCAGCCAGCTCAGAATCAC	AGGTCTGATGTCCCAGAAACACC
BSG	CTTGAATGACAGCGCCACAGAG	TCGTCGGAGTCCACCTTGAAC
CCL5	TGCTGCTTTGCCTACATTGCC	TTTCGGGTGACAAAGACGACTG
CTSB	TGACTGGGGTGACAATGGCTTC	TTCCAGCCACCACTTCTGATTC
CTSL	ATCACAGTTTAGAGGCACAGTGG	CCTCCATCCTTCTTCATTCATGCC
CXCL8	TCTTCATTGACCAAGGAAATCGGC	TATGGTCCGGGGTGCATTATCTCTA
CXCL10	GTGGCATTCAAGGAGTACCTCTC	CGTGGACAAAATTGGCTTGCAG
CYP11A1	AGACCTGGAAGGACCATGTG	TCCTCGAAGGACATCTTGCT
CYP17A1	GTGGAGACCACCACCTCTGT	GCTGAAACCCACATTCTGGT
CYP19A1	CCAGGTCCTGGCTACTGCAT	GATCCCCATCCACAGGAATCT
FSHR	TGCCATTGAACTGAGGTTTG	GGTCCCCAAATCCTGAAAT
FURIN	ATGGGACGCTGACCAAGTTCAC	AAGCCTTCCTCGCACACCACAC
HKU-ORF1b-nsp14	TGGGGTTTTACAGGTAACCT	AACACGCTTAACAAAGCACTC
HSD17B3	TCCTTGGCCTCTCTACTCCA	AGACAGCATATGGGGTCAGC
HSD3B2	GCCTGTTGGTGGAAGAGAAG	GCAGGCTCTTTTCAGGAATG
IFIT1	CTTGAGCCTCCTTGGGTTCGTC	GTTCTCAAAGTCAGCAGCCAGTC
IFITM1	CCGTGAAGTCTAGGGACAGGAAG	CACAGAGCCGAATACCAGTAACAG
IFNb	CAGAAGCTCCTGTGGCAATTG	TCCTCAGGGATGTCAAAGTTCA
IL1b	CACGATGCACCTGTACGATCAC	ACATGGAGAACACCACTTGTTGC
IL6	ACACAGACAGCCACTCACCTC	TGTTTTCTGCCAGTGCCTCTTTG
IL18	AGAAGTTTTTGAAGAGGGCTGAGA	AGATTTGCTTGAAGTTTCACTGGCA
INHBB	AGCCTCCAGGATACCAGCAA	TCTCCGACTGACAGGCATTTG
INSL3	GCGACCGTGAGTTGCTACAG	GGTCCCAGCGTGAGATTACTG
ISG15	CGAACCTCTGAGCATCCTGGTG	CCTCGAAGGTCAGCCAGAACAG
LGP2	CCCACCATGTCAATGTGAACCC	CCTTGAAGACTTTGTTGATGACCAC
LHCGR	GCCGGTCTCACTCGACTATC	TCATTAAGTCCTCTGAAAGCTTGA
MDA5	AAAGCACTGCAAAAGAAGTGTGC	TGCACCATCATTGTTCCCCAAG
MX1	CGTTAGCCGTGGTGATTTAGCAG	ATCCTTCAATCCCGCCAGCTC
NRP1	ACCTTGGTGGGATTGCTGTGG	GTATCCTGGCGTGCTCCCTGTTTC
OAS1	CATGTGCTGCCTGCCTTTCATG	TCGATGAGCTTGACATAGATTTGGG
PGK2	ACCAGAGGATCAAGGCTTCC	TCAGGCCGACCTAGATGACT
PLZF	CTCATTCAGCGGGTGCCAAAG	AGTATGGGTCTGCCTGTGTGTC
PRM2	GCAGAAGGCGCAAAAGAC	TCTGCATGTTCTCTTCCTGGT
RHOX	CACGAAGTTCACGCTGTTGC	TTCGGCAAGTTCCCTTCTTGTG
RIGI	GACACAGAGAGTCTGGCAAAGAG	CTTTGTCTGGCATCTGGAACACC
RSAD2	ACATGACGGAACAGATCAAAGCAC	AGCATCTTCTCCACAATTCTCACC
SRD5A3	AGGAATGCCTACATAACAGGG	CTCCAAATGGGATCCTGTGGT
SOCS3	TAAGGGGTAAAGGGCGCAAAGG	CCTGGTTGGCTTCTTGTGCTTG
STAR	GGCTGGCATGGCCACAGAC1	TTGGGCAGCCACCCCTTGA
TIM1	AGAAACCCACCCTACGACACTG	CGGTGTCATTCCCATCTGTTGTG
TLR2	GACCGCAATGGTATCTGCAAGG	CTGTTGTTGGACAGGTCAAGGC
TLR3	ACTGAACCATGCACTCTGTTTGC	AAGGCACCTATCCGTTCTTTCTG
TMPRSS2	TATCGACAAATGAGGGCAGACG	TCATGCACGGGGAAGCAAAAC
TNFa	CTGTAGCCCATGTTGTAGCAAACC	TCTCTCAGCTCCACGCCATTG
TSPO	GGCTTCACAGAGAAGGCTG	ACTGACCAGCAGGAGATCCA
USP18	ACTCCTTGATTTGCGTTGAC	TTTCCCACGGGTCTTCTT
ZO-1	TTATTTGGGCTGTGGCGTGAG	CCTCCATTGCTGTGCTCTTGG

Suppl Table 2

Supplementary Table 2. Antibodies used for immunostainings.

Antigen	Isotype, clone	Company, order	Concentration	Demasking buffer	Blocking buffer
αSMA	Mouse IgG2a, #1A4	Dako, #M0814	1.7 µg/mL	Citrate pH6	PBS 10% BSA
ACE2	Goat IgG, polyclonal	R&D, #AF933	5 µg/mL	Citrate pH6	PBS 10% BSA
Cleaved Caspase 3	Rabbit IgG, polyclonal	Cell Signaling, #9661	0.35 µg/mL	EDTA pH8	TBS 10% BSA
CD163	Mouse IgG1, #10D6	Leica, #NCL-L-CD163	0.5 µg/mL	Citrate pH6	PBS 10% BSA
CD68	Mouse IgG1, #KP1	Dako, #M0814	1.85 µg/mL	Citrate pH6	PBS 10% BSA
CYP11A1	Rabbit IgG, polyclonal	Sigma #HPA016436	0.5 µg/mL	Citrate pH6	PBS 10% BSA
DDX4 (for IHC)	Rabbit IgG, polyclonal	Abcam, #ab13840	2 µg/mL	Citrate pH6	PBS 2% BSA
DDX4 (for RNAscope)	Rabbit IgG, polyclonal	Sigma, #HPA037764	1 µg/mL	Citrate pH6	TBS 10% Chicken serum
dsRNA	Mouse IgG2ak, #J2	Scicons, #RNT-SCI-10010200	1 µg/mL	Citrate pH6	TBS 10% Chicken serum
SARS-NP	Mouse IgG2b, #3851	Genetex, #GTX36802	1 µg/mL	Citrate pH6	PBS 10% BSA
TMPRSS2	Rabbit IgG, #EPR3862	Abcam, #ab109131	0.5 µg/mL	EDTA pH9	TBS 10% Chicken serum
Vimentin (for IHC)	Rabbit IgG, #EPR3776	Abcam, #ab92547	0.78 µg/mL	Citrate pH6	Ø
Vimentin (for RNAscope)	Rabbit IgG, polyclonal	Sigma, #HPA001762	1 µg/mL	Citrate pH6	TBS 5% BSA
ZO1	Mouse IgG1, #1A12	Invitrogen #33-9100	1 µg/mL	EDTA pH8	Ø
